# Clinical Problem-Solving: A 19-Year-Old Woman With Progressive Neurological Decline and Multiple Intracranial Lesions

**DOI:** 10.1177/19418744241273283

**Published:** 2024-08-13

**Authors:** Rumyar Ardakani, Kimmo Hatanpaa, Yanel De Los Sanotos, Paula Hardeman, Lauren Tardo

**Affiliations:** 1 Department of Neurology, 129263 University of Colorado Anschutz Medical Campus, Aurora, CO, USA; 2 Department of Pathology, 12334 UT Southwestern Medical Center, Dallas, TX, USA; 3 Department of Neurology, 12334 UT Southwestern Medical Center, Dallas, TX, USA

**Keywords:** Balo’s concentric sclerosis, demyelination, multiple sclerosis, tumefactive demyelination

## Abstract

The differential diagnosis for multiple intracranial lesions in a young adult is broad and includes demyelinating, neoplastic, and infectious etiologies. In this report, we describe the case of a 19-year-old immunocompetent woman presenting with progressive headaches and aphasia. MRI of the brain revealed multiple, large supratentorial lesions with concentric bands of alternating T2 signal intensities and peripheral contrast enhancement. Cerebrospinal fluid (CSF) analysis was overall bland with negative oligoclonal bands. Serum antibody testing for neuromyelitis optica (NMO) and myelin-oligodendrocyte associated disease (MOGAD) were negative. A broad infectious work-up was also unrevealing. A definitive diagnosis was ultimately obtained after brain biopsy and the patient was started on appropriate therapy. This case highlights a diagnostic framework in evaluating immunocompetent patients presenting with multiple intracranial lesions and progressive neurological decline. The main differential diagnoses for this constellation of radiological and clinical findings are discussed and a literature review is performed on the revealed diagnosis. Lastly, both acute and long-term therapeutic approaches are reviewed.

## Section 1

A 19-year-old woman presented with a three-week history of progressive headaches. The headaches began shortly after a sinus infection and were associated with photophobia, nausea, and vomiting. She subsequently developed progressive word-finding difficulties and increasing somnolence. The patient denied recent fever, chills, weight loss, substance use, or prior neurological symptoms. Medical history was notable only for occasional sinus infections. She was not taking any medications or supplements. There was no family history of similar presentations. She had no prior surgical history.

On examination, the patient was afebrile and hemodynamically stable. She was lethargic with a moderate receptive aphasia. She was unable to follow simple or complex commands. Cranial nerve examination was normal. She was able to lift all extremities anti-gravity without apparent asymmetry. She had brisk reflexes throughout both upper and lower extremities with mute plantar responses.

**Figure 1.**
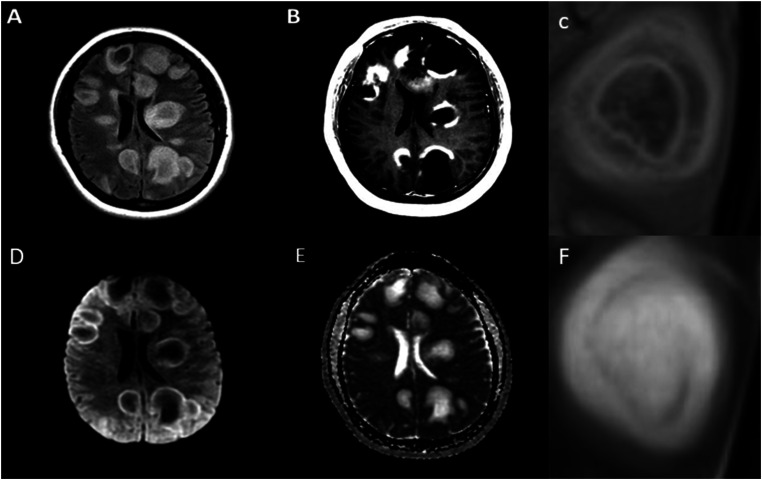
T2-weighted imaging (A) showing multiple supratentorial lesions consisting of concentric bands of alternating T2 signal intensities. T1 post-contrast imaging (B) shows peripheral ring enhancement with ‘open ring’ pattern in several lesions. Magnified T2 FLAIR imaging (C) demonstrating closer view of concentric banding. Diffusion weighted imaging (D) and apparent diffusion coefficient imaging (E) demonstrate restricted diffusion in the outer ring of several lesions. Magnified T2 weighted imaging (F) showing closer view of concentric banding.

Brain MRI was obtained shortly after presentation and is demonstrated in Figure 1.

Questions for Consideration

1. How would you describe and classify the lesions demonstrated on the brain MRI?
2. What is your differential diagnosis based on the clinical presentation and imaging findings so far?

## Section 2

The brain MRI reveals multiple T2-hyperintense lesions in the bilateral supratentorial white matter of varying sizes with mild surrounding vasogenic edema. The largest lesions are approximately 4-5 centimeters in diameter. Some lesions demonstrate irregular, concentric bands of alternating T2 signal intensities. Contrasted imaging reveals a predominately peripheral pattern of enhancement with some lesions demonstrating an “open ring” pattern. There is a mild degree of diffusion restriction in the outer ring of some lesions. Given this pattern of radiological findings and size of lesions, these lesions can be classified as likely tumefactive demyelinating lesions.

The differential diagnosis for this type of presentation is broad. Given the appearance of imaging findings and patient’s age, demyelinating conditions such as multiple sclerosis, neuromyelitis optica (NMO), myelin-oligodendrocyte glycoprotein antibody-associated disease (MOGAD), and acute disseminated encephalomyelitis (ADEM) should be considered. Although this imaging pattern is not seen in typical multiple sclerosis, it may be seen in more atypical variants of multiple sclerosis including Marburg variant and Balo’s concentric sclerosis (BCS).^1^

In addition to demyelinating conditions, neoplastic etiologies should also be considered in the differential diagnosis. CNS lymphoma and glioblastomas may result in a similar imaging pattern but would typically present with more vasogenic edema and mass effect. Lastly, infectious causes such as pyogenic abscesses, tuberculomas, and CNS toxoplasmosis should be included in the differential. However, the absence of constitutional symptoms and immunocompetence of the patient made this less likely.

Questions for consideration:

1. What other studies should you perform based on your differential diagnosis?
2. How will these studies change your differential diagnosis?

## Section 3

Routine lab work-up including complete blood count with differential and comprehensive metabolic panel were both normal. Cerebrospinal fluid (CSF) analysis revealed 1 WBC/µL, 1 RBC/µL, normal protein (21 mg/dL), 0 oligoclonal bands, and negative bacterial, fungal, and mycobacterial cultures. Both CSF flow and cytology were negative with no evidence of aberrant cell populations; however, the sample was limited by hypocellularity. Serum QuantiFERON-TB Gold testing for tuberculosis also returned negative. Serum aquaporin-4 (AQP4) IgG antibody and myelin-oligodendrocyte glycoprotein (MOG) IgG antibody eventually also returned negative. Serum LDH was not elevated. Spinal cord imaging including cervical and thoracic spine revealed no concerning areas of cord signal change. CT chest, abdomen, and pelvis was also unremarkable. PET scan was not available inpatient.

Given negative serological testing for NMO and MOG, it was felt that these two diagnoses were less likely. Given the absence of CSF oligoclonal bands, multiple sclerosis was also felt to be less likely, though not fully excluded as oligoclonal bands are not as frequently detected in atypical variants of MS. Lastly, the suspicion for an infectious process continued to remain low based on the patient’s presentation and infectious lab work-up.

As neoplastic etiologies remained on the differential, magnetic resonance spectroscopy (MRS) of the brain was pursued. This revealed elevated choline peak, decreased N-acetylaspartate (NAA) peak, and elevated lactate peak. These findings were ultimately felt to be non-specific and did not entirely rule out a neoplastic process. Therefore, the main differential at this point primarily included atypical variants of multiple sclerosis and neoplastic processes – particularly CNS lymphoma.

Questions for consideration:

1. What would be your next step in management and treatment of this patient?
2. If the patient fails to adequately respond to your first-line choice of treatment, what additional work-up or treatment would you perform?

## Section 4

**Figure 2.**
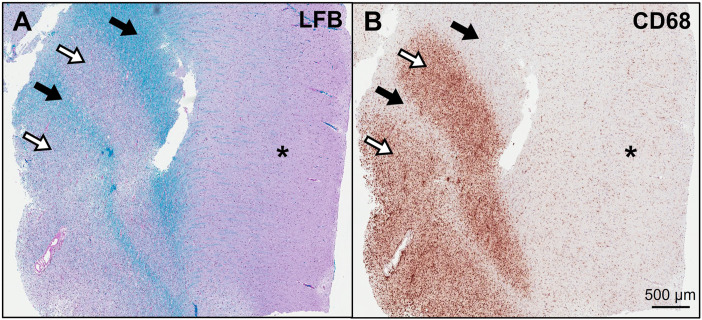
(A) A Luxol fast blue (LFB) stain for myelin (blue) reveals alternating bands of demyelination (open arrows) and preserved myelin (solid arrows) in the subcortical white matter. The overlying cortex is denoted by an asterisk. (B) A CD68 immunohistochemical stain for macrophages (brown) highlights dense inflammatory infiltrates in a similar band-like distribution, indicating active demyelination.

Since atypical demyelinating syndromes and CNS lymphoma remained highest on the differential, it was decided to pursue empiric treatment with high-dose steroids. The patient received 5 doses of IV methylprednisolone 1000 mg daily over 5 days with minimal clinical improvement. Therefore, plasma exchange was pursued and she completed a total of 5 sessions with only modest improvement in mental status and wakefulness. Given persistent symptoms and diagnostic uncertainty, it was decided to pursue a brain biopsy for a definitive diagnosis. Biopsy was completed approximately 2 weeks after completion of pulse steroids. Biopsy of a right frontal lobe lesion demonstrated alternating rings of demyelinated and myelinated axons in an “onion bulb” pattern (Figure 2). Based on these findings, the patient was diagnosed with Balo’s concentric sclerosis. Given severity of disease burden, she received an induction dose of cyclophosamide 1000 mg/m^2^and was discharged with a prolonged steroid taper. She had excellent recovery over the following months with very little residual deficits other than occasional mild word-finding difficulty. At outpatient follow-up, it was decided to start rituximab 1000 mg every six months for maintenance immunosuppressive therapy to prevent disease relapse. She is now two years out from her acute presentation and continues on rituximab. At this time, she has no residual deficits. MRI brain has demonstrated continued improvement in the extensive white matter lesions with no residual mass effect.

## Discussion

Balo’s concentric sclerosis is a rare demyelinating disease of the central nervous system.^2^ The typical presentation involves acute or subacute progressive neurological decline with specific symptoms often depending on the location and size of white matter lesions.^3^ Typical presentations can include headache, focal weakness, sensory changes, ataxia, dysarthria, aphasia, and diplopia.^2,3^The hallmark of the disease is the presence of concentric and multi-layered “ring-like” lesions in the cerebral white matter. This can be appreciated best on MRI brain as concentric bands of varying T1 and T2 intensities.^4^ The presence of peripheral open-ring enhancement can be another sign suggestive of the diagnosis. These findings correlate pathologically with concentric layers of alternating myelination and demyelination under tissue microscopy.^3^

The imaging findings in Balo’s concentric sclerosis can mimic other non-demyelinating conditions including neoplastic and infectious processes. In our case, we did not have a high suspicion for an infectious process due to the absence of constitutional symptoms and the immunocompetent status of our patient. However, a neoplastic process such as CNS lymphoma remained high on our differential prior to brain biopsy. It should also be noted that the relative steroid responsiveness of both CNS lymphoma and demyelinating disease can also pose a diagnostic challenge and may impact the diagnostic yield of a biopsy. Although the MRS findings were not definitive for a particular diagnosis, the findings are consistent with previously reported findings in Balo’s concentric sclerosis.^5^

While Balo’s concentric sclerosis is recognized as an atypical demyelinating disease, there is ongoing controversy whether it is a phenotypical subtype of multiple sclerosis or a separate disease entity with a distinct pathophysiology.^6^ The two conditions share significant overlap in both clinical, radiologic, and pathologic features.^2^ Compared to multiple sclerosis, however, patients with Balo’s concentric sclerosis are less likely to have positive CSF oligoclonal bands and are more likely to have a monophasic and self-limited disease course.^7^ Of note, some patients presenting with Balo’s concentric sclerosis will demonstrate concurrent MS-like brain lesions and positive oligoclonal bands. Patients with these findings are more likely to develop a relapsing-remitting disease course similar to multiple sclerosis.^6,7^

The first-line treatment for Balo’s concentric sclerosis typically consists of high-dose intravenous steroids often followed by a steroid taper over several weeks to months.^8^ Although the data is scarce due to the rarity of the condition, plasma exchange is an appropriate second-line option for patients who do not adequately respond to high-dose intravenous steroids given its successful track record in other acute CNS inflammatory demyelinating disease.^2,9^ Other second-line options may include intravenous immunoglobulins, rituximab, and cyclophosphamide.^2,8^ It is controversial whether long-term immunosuppressive therapy should be initiated in patients with the condition. In patients who meet criteria for relapsing-remitting multiple sclerosis, it is reasonable to pursue long-term immunotherapy with high-efficacy medications used in multiple sclerosis.^1,2,8^ However, the decision to initiate maintenance immunotherapy in other patients should be made on a case-by-case basis with consideration of patient preference.

In conclusion, Balo’s concentric sclerosis should be considered in the differential diagnosis for tumefactive demyelinating lesions. Imaging findings may mimic other demyelinating conditions, neoplasms such as CNS lymphoma, or infectious processes. Brain biopsy is often needed for definitive diagnosis.
